# Mindfulness-based interventions and employment: Descriptive analysis of the MER-ACT project

**DOI:** 10.1192/j.eurpsy.2022.1793

**Published:** 2022-09-01

**Authors:** T. Castellanos Villaverde, G. Navarro Oliver, I. Torrea Araiz, E. Vidal Bermejo, A. Hospital Moreno, I. Louzao Rojas, E. Fernández-Jiménez

**Affiliations:** Hospital Universitario La Paz, Department Of Psychiatry, Clinical Psychology And Mental Health, Madrid, Spain

**Keywords:** mental health, Employment, group intervention, Mindfulness

## Abstract

**Introduction:**

Evidence shows unemployment as a negative impact factor on a variety of health outcomes. Regarding mental health, unemployment is considered one of the most consolidated risk factors for morbidity. This relationship is considered bi-directional. Prevention and wellness promotion are essential guidelines for mental health providers.

**Objectives:**

To describe the work status in a sample of patients with anxiety disorders after two types of group mindfulness-based interventions in the MER-ACT project.

**Methods:**

A descriptive analysis was conducted on work status before and 6 months after two types of mindfulness-based interventions. The group treatments were Acceptance and Commitment Therapy and a Mindfulness-based Emotional Regulation intervention, during 8 weeks, guided by two Clinical Psychology residents. The employment change was calculated (percentage of change from unemployed or temporary incapacity to employed).

**Results:**

The work status of participants of the sample (n = 40), before and 6 months after interventions, were employed: 55% vs. 60%; temporary incapacity: 12.5% vs. 12.5%; unemployed: 25% vs. 20% and others: 7.5% vs. 7.5%. In the same period, the unemployment rate in the Spanish general population was from 13.8% to 14.5%. After 6 months the percentage of change on work status was 25% (15% improved their employment situation).

**Conclusions:**

Preliminary results show worse work status of participants compared to the Spanish general population. It is recommendable to include well-established risk factor measurements to establish the effectiveness of interventions in mental health. More research is required to determine the impact of interventions on the employment status.

**Disclosure:**

No significant relationships.

